# Risk of Violent Crime in Individuals with Epilepsy and Traumatic Brain Injury: A 35-Year Swedish Population Study

**DOI:** 10.1371/journal.pmed.1001150

**Published:** 2011-12-27

**Authors:** Seena Fazel, Paul Lichtenstein, Martin Grann, Niklas Långström

**Affiliations:** 1 Department of Psychiatry, University of Oxford, Warneford Hospital, Oxford, United Kingdom; 2 Centre for Violence Prevention, Karolinska Institutet, Stockholm, Sweden; 3 Department of Medical Epidemiology and Biostatistics, Karolinska Institutet, Stockholm, Sweden; 4 Swedish Prison and Probation Service, Norrköping, Sweden; University of Western Sydney, Australia

## Abstract

**Background:**

Epilepsy and traumatic brain injury are common neurological conditions, with general population prevalence estimates around 0.5% and 0.3%, respectively. Although both illnesses are associated with various adverse outcomes, and expert opinion has suggested increased criminality, links with violent behaviour remain uncertain.

**Methods and Findings:**

We combined Swedish population registers from 1973 to 2009, and examined associations of epilepsy (*n* = 22,947) and traumatic brain injury (*n* = 22,914) with subsequent violent crime (defined as convictions for homicide, assault, robbery, arson, any sexual offense, or illegal threats or intimidation). Each case was age and gender matched with ten general population controls, and analysed using conditional logistic regression with adjustment for socio-demographic factors. In addition, we compared cases with unaffected siblings.

Among the traumatic brain injury cases, 2,011 individuals (8.8%) committed violent crime after diagnosis, which, compared with population controls (*n* = 229,118), corresponded to a substantially increased risk (adjusted odds ratio [aOR] = 3.3, 95% CI: 3.1–3.5); this risk was attenuated when cases were compared with unaffected siblings (aOR = 2.0, 1.8–2.3). Among individuals with epilepsy, 973 (4.2%) committed a violent offense after diagnosis, corresponding to a significantly increased odds of violent crime compared with 224,006 population controls (aOR = 1.5, 1.4–1.7). However, this association disappeared when individuals with epilepsy were compared with their unaffected siblings (aOR = 1.1, 0.9–1.2). We found heterogeneity in violence risk by age of disease onset, severity, comorbidity with substance abuse, and clinical subgroups. Case ascertainment was restricted to patient registers.

**Conclusions:**

In this longitudinal population-based study, we found that, after adjustment for familial confounding, epilepsy was not associated with increased risk of violent crime, questioning expert opinion that has suggested a causal relationship. In contrast, although there was some attenuation in risk estimates after adjustment for familial factors and substance abuse in individuals with traumatic brain injury, we found a significantly increased risk of violent crime. The implications of these findings will vary for clinical services, the criminal justice system, and patient charities.

*Please see later in the article for the Editors' Summary*

## Abstract 2

Seena Fazel and colleagues report findings from a longitudinal follow-up study in Sweden that evaluated the risks of violent crime subsequent to hospitalization for epilepsy, or traumatic brain injury. The researchers control for familial confounding with sibling controls. The analyses call into question an association between epilepsy and violent crime, although they do suggest that there may be a relationship between traumatic brain injury and violent crime.

## Editors' Summary

**Background:**

News stories linking mental illness (diseases that appear primarily as abnormalities of thought, feeling or behavior) with violence frequently hit the headlines. But what about neurological conditions—disorders of the brain, spinal cord, and nerves? People with these disorders, which include dementia, Parkinson's disease, and brain tumors, often experience stigmatization and discrimination, a situation that is made worse by the media and by some experts suggesting that some neurological conditions increase the risk of violence. For example, many modern textbooks assert that epilepsy—a neurological condition that causes repeated seizures or fits—is associated with increased criminality and violence. Similarly, various case studies have linked traumatic brain injury—damage to the brain caused by a sudden blow to the head—with an increased risk of violence.

**Why Was This Study Done?:**

Despite public and expert perceptions, very little is actually known about the relationship between epilepsy and traumatic brain injury and violence. In particular, few if any population-based, longitudinal studies have investigated whether there is an association between the onset of either of these two neurological conditions and violence at a later date. This information might make it easier to address the stigma that is associated with these conditions. Moreover, it might help scientists understand the neurobiological basis of violence, and it could help health professionals appropriately manage individuals with these two disorders. In this longitudinal study, the researchers begin to remedy the lack of hard information about links between neurological conditions and violence by investigating the risk of violent crime associated with epilepsy and with traumatic brain injury in the Swedish population.

**What Did the Researchers Do and Find?:**

The researchers used the National Patient Register to identify all the cases of epilepsy and traumatic brain injury that occurred in Sweden between 1973 and 2009. They matched each case (nearly 23,000 for each condition) with ten members of the general population and retrieved data on all convictions for violent crime over the same period from the Crime Register. They then linked these data together using the personal identification numbers that identify Swedish residents in national registries. 4.2% of individuals with epilepsy had at least one conviction for violence after their diagnosis, but only 2.5% of the general population controls did. That is, epilepsy increased the absolute risk of a conviction for violence by 1.7%. Using a regression analysis that adjusted for age, gender, and various socio-demographic factors, the researchers calculated that the odds of individuals with epilepsy committing a violent crime were 1.5 times higher than for general population controls (an adjusted odds ratio [aOR] of 1.5). The strength of this association was reduced when further adjustment was made for substance abuse, and disappeared when individuals with epilepsy were compared with their unaffected siblings (a sibling control study). Similarly, 8.8% of individuals with traumatic brain injury were convicted of a violent crime after their diagnosis compared to only 3% of controls, giving an aOR of 3.3. Again, the strength of this association was reduced when affected individuals were compared to their unaffected siblings (aOR = 2.0) and when adjustment was made for substance abuse (aOR = 2.3).

**What Do These Findings Mean?:**

Although some aspects of this study may have affected the accuracy of its findings, these results nevertheless challenge the idea that there are strong direct links between epilepsy and violent crime. The low absolute rate of violent crime and the lack of any association between epilepsy and violent crime in the sibling control study argue against a strong link, a potentially important finding given the stigmatization of epilepsy. For traumatic brain injury, the reduced association with violent crime in the sibling control study compared with the general population control study suggests that shared familial features may be responsible for some of the association between brain injury and violence. As with epilepsy, this finding should help patient charities who are trying to reduce the stigma associated with traumatic brain injury. Importantly, however, these findings also suggest that some groups of patients with these conditions (for example, patients with head injuries who abuse illegal drugs and alcohol) would benefit from being assessed for their risk of behaving violently and from appropriate management.

**Additional Information:**

Please access these websites via the online version of this summary at http://dx.doi.org/10.1371/journal.pmed.1001150.

- This study is further discussed in a *PLoS Medicine* Perspective by Jan Volavka
- The US National Institute of Neurological Disorders and Stroke provides detailed information about traumatic brain injury and about epilepsy (in English and Spanish)
- The UK National Health Service Choices website provides information about severe head injury, including a personal story about a head injury sustained in a motor vehicle accident, and information about epilepsy, including personal stories about living with epilepsy
- Healthtalkonline has information on epilepsy, including patient perspectives
- MedlinePlus provide links to further resources on traumatic brain injury and on epilepsy (available in English and Spanish)

## Introduction

Despite evidence demonstrating an association between certain severe mental illnesses and violence [1],[2], much less is known about the relationship of neurological disorders with violent and other antisocial behaviour [3],[4]. Despite this, expert opinion has suggested that some neurological conditions increase the risk of violence. Reviews and modern textbooks assert that epilepsy is associated with violence risk [3],[5]–[8], a view widely held in the 19th century [9]. However, a recent systematic review suggested caution in drawing any conclusions about the relationship between epilepsy and violence [10], as the small evidence base is based on prisoner samples [11],[12] or individuals with childhood epilepsy [13]. For traumatic brain injury, the theoretical basis for an association with violence is much stronger. Influential case reports, such as that for Phineas Gage [14], case–control studies of war veterans who experienced frontal lobe damage [15], and case series of murderers [16] and brain injured prisoners [17] have provided some support. On the other hand, there is little evidence of causal mechanisms, and there are, for example, socio-economic differences between head injured persons and others [18]. Yet, as with epilepsy, a recent review found no published population-based or longitudinal surveys [10], and the potential role of injuries incurred in childhood is unknown [19].

There are a number of reasons why examining the association of neurological disorders with violence is potentially considerable. First, considerable stigma is associated with epilepsy [20] and traumatic brain injury [21], and accurate information on risk for adverse outcomes could be relevant in addressing this. Second, it may provide information on mechanisms underlying violence, assisting the understanding of the neurobiological basis of violent behaviour. Third, information on the prevalence and relative risks of violence and criminality could inform neurology, emergency medicine, rehabilitation medicine, and general and forensic psychiatric services in determining when violence risk assessment and management might be most valuable, based on the specific neurological disorder a patient has. Finally, the public health impact of associations between these two common neurological conditions and violence is potentially important. The prevalence of hospitalised traumatic brain injury is around 8 million in Europe [22] (0.2%–0.3% of the general population), and 0.5% of the population is estimated to suffer from epilepsy [23]. Furthermore, brain injury is one of the most common chronic conditions among prisoners [24], and the most frequent reason for presentation of individuals to emergency departments [25]. Thus, understanding associations and mechanisms could assist in improved risk assessment and management of the large numbers of individuals presenting to health services with these two disorders. In the present study, we used longitudinal total population designs to examine the relationship of epilepsy and traumatic brain injury with violent crime in Sweden from 1973 until 2009.

## Methods

The Regional Ethics Committee at the Karolinska Institutet approved the study (2009/939-31/5). Data were merged and anonymized by an independent government agency (Statistics Sweden), and the code linking the personal identification numbers to the new case numbers was destroyed immediately after merging. Therefore, informed consent was not required.

### Study Setting

We linked several longitudinal, nationwide population-based registries in Sweden: the National Patient Register (held at the National Board of Health and Welfare), the Crime Register (National Council for Crime Prevention), the National Censuses from 1970 and 1990 (Statistics Sweden), and the Multi-Generation Register (Statistics Sweden). The Multi-Generation Register connects each person born in Sweden in 1933 or later and ever registered as living in Sweden after 1960 to their parents [26]. For immigrants, similar information exists for those who became citizens of Sweden before age 18 y, together with one or both parents. In Sweden, all residents including immigrants have a unique ten-digit personal identification number that is used in all national registers, thus making the linking of data in these registers possible. We restricted the population to individuals born between 1958 and 1994, so that all individuals who were at least 15 y (the age of criminal responsibility in Sweden) were included from 1973 onwards, to the end of follow-up in 2009 (*n* = 5,665,112).

### Individuals with Neurological Disorder

We identified cases with epilepsy or traumatic brain injury from the National Patient Register, which includes individuals admitted to any hospital (since 1973) or having outpatient appointments (since 2001) in Sweden [27]. Cases with epilepsy had to have at least two separate patient episodes according to International Classification of Diseases (ICD) ICD-8 (1973–1986; diagnostic codes 345.00–345.99), ICD-9 (1987–1996; codes 345J, K, L, M, N, P, Q, W, X), or ICD-10 (from 1997 onwards; codes G40.1–G40.9, G41). We decided that epilepsy had to be diagnosed at two separate occasions to increase diagnostic precision by minimising false positive diagnoses; hence, those with only one diagnosis were excluded (*n* = 22,084). For traumatic brain injury (not including concussion), we selected cases based on one or more patient episodes according to ICD-8 (1973–1986; diagnostic codes 851–852), ICD-9 (1987–1996; codes 851–854), or ICD-10 (from 1997 onwards; codes S06.01–S06.09). We also investigated comorbidity with drug and alcohol use disorders. Data were also extracted for every individual on all inpatient (1973–2009) and outpatient (2001–2009) diagnoses with principal or comorbid diagnoses of alcohol abuse or dependence (ICD-8: 303; ICD-9: 303, 305.1; ICD-10: F10, except x.5) or drug abuse or dependence (ICD-8: 304; ICD-9: 304, 305.9; ICD-10: F11–F19, except x.5). This information was used as a marker for comorbid alcohol and/or drug abuse disorders.

### Diagnostic Validity

Swedish patient register data on diagnoses have good to excellent validity for a range of conditions, such as acute myocardial infarction [28], injuries [29], acute stroke [30], Guillain-Barré syndrome [31], and schizophrenia [32],[33]. Overall, the positive predictive value of the inpatient register, in a recent review, was found to be 85%–95% for most diagnoses [34]. Little information exists on diagnostic validity of comorbid conditions in neurological disorders. However, fair to moderate agreement for comorbid substance abuse has been found in schizophrenia (κ of 0.37, standard error = 0.23, *p*<0.001, corresponding to 68% full agreement) [35]. Only around 1% of hospital admissions have missing personal identification numbers [36]. Consequently, the register has been used in a variety of epidemiological investigations [35],[37].

### Control Populations

For each disorder, ten general population individuals without the specific patient register diagnosis of the cases were matched individually to cases by birth year and gender.

We also conducted the following subanalyses. In the epilepsy group, we separately analysed cases first diagnosed as adults (i.e., aged 16 y and over) with those diagnosed earlier to examine whether there is a difference in risk between childhood-onset and adult-onset epilepsy. This was done because a previous study suggested an inverse relationship between childhood-onset epilepsy and juvenile delinquency [13]. We investigated categories of epilepsy and classified them into four types according to the diagnosis at second admission, as in previous work [38]: complex partial seizures (ICD-8: 345.31; ICD-9: 345M; ICD-10: G40.2), other partial seizures (ICD-8: 345.30, 345.38, 345.39; ICD-10: G40.0, G40.1), generalised epilepsy (ICD-8: 345.09, 345.10, 345.11; ICD-9: 345J, 345K; ICD-10: G40.3), and other or unspecified epilepsy (ICD-8: 345.18, 345.19, 345.29, 345.99; ICD-9: 345L, 345P, 345Q, 345W, 345X; ICD-10: G40.4, G40.5, G40.6, G40.7, G40.8, G40.9, G41). As an index of severity, we compared those whose first treatment episode lasted for 15 d or more (90th percentile) with the others.

In the traumatic brain injury group, we conducted stratified analyses by age of onset, diagnostic subgroup, and severity. Specifically, we compared individuals with adult-onset traumatic brain injury (i.e., aged 16 and over at the onset of disease) with those with childhood-onset traumatic brain injury. We restricted subgroup analyses to ICD-10 diagnoses of traumatic brain injury (comparable subgroups are not found in ICD-8/9). For this, we subdivided those with traumatic brain injury into: (a) traumatic cerebral oedema (S06.1) and diffuse brain injury (S06.2), (b) focal brain injury (S06.3), and (c) epidural, traumatic subdural, or subarachnoid haemorrhage (S06.4–6). In addition, we compared rates of violent offending in individuals with diagnoses of concussion (ICD-8/9: 850; ICD-10: S06.0)—a less severe form of brain injury—with traumatic brain injury.

### Sibling Control Studies

For both diagnoses, we conducted additional analyses using unaffected full siblings of cases as controls. Using the Multi-Generation Register, we identified as cases individuals with epilepsy (*n* = 10,360) who also had full siblings without epilepsy, and those persons with traumatic brain injury (*n* = 11,499) who also had full siblings without traumatic brain injury. These individuals were compared with their unaffected full siblings (*n* = 17,448 full sibling controls compared to *n* = 10,360 individuals with epilepsy; *n* = 19,628 full sibling controls compared to *n* = 11,499 cases with traumatic brain injury). We conducted this additional analysis because the possibility of residual confounding was considered high, particularly in traumatic head injury [39], with impulsivity being a possible mechanism [40]. For these analyses, we adjusted by gender and age.

### Outcome Measures

Data on all convictions for violent crime from 1 January 1973 to 31 December 2009 were retrieved for all individuals aged 15 y and older (15 y is the age of criminal responsibility in Sweden; antisocial behaviour under this age is not prosecuted or systematically registered). Consistent with other work in schizophrenia and severe mental illness, violent crime was defined as homicide, assault, robbery, arson, any sexual offense (rape, sexual coercion, child molestation, indecent exposure, and sexual harassment), or illegal threats or intimidation [36]. Attempted and aggravated forms of included offenses, where applicable according to the Swedish Criminal Code, were also included. Burglary, other property offenses, and traffic and drug offences were excluded. In individuals born from 1954 to 1994, this amounted to 217,134 (unique) persons with at least one violent conviction.

Conviction data were used because the Criminal Code in Sweden determines that individuals are convicted as guilty regardless of medical conditions (such as epileptic automatisms) or mental disorder (which may be comorbid with neurological conditions). Therefore, it includes also those who are found not guilty by reason of insanity (who would be acquitted in other jurisdictions), those receiving custodial or non-custodial sentences, and individuals transferred to psychiatric hospitals on sentencing. Furthermore, conviction data included those cases where the prosecutor decided to caution or fine. In addition, as plea-bargaining is not permitted in Sweden, conviction data accurately reflect the extent of officially resolved criminality. The Crime Register has excellent coverage; only 0.05% of crimes had incomplete personal identification numbers during 1988–2000 [36].

### Socio-Demographic Covariates

Household income (divided into thirds) of the family of origin for those 15 y or younger at the time of the 1990 census was used as a proxy for income. Single marital status was defined as being unmarried. Immigrant status was defined as being born outside of Sweden. Missing data were not replaced by imputation or other methods.

### Analyses

Only violent convictions recorded after first diagnosis for traumatic brain injury and epilepsy were included. We estimated the association between having been diagnosed with either of these neurological disorders and violent offending with conditional logistic regression, as per related work using matched or sibling controls [35], using the clogit command in Stata, version 10 (StataCorp). The clogit command fits conditional (fixed effects) logistic regression models to matched case–control groups. Ten controls from the general population were selected for each case and matched by birth year and gender. In the sibling control study, all unaffected siblings were compared with their sibling with traumatic brain injury or epilepsy, and analyses were adjusted for age and gender. Among the general population and sibling controls, violent crime was counted only if it occurred after the date of diagnosis in the matched cases. We included three confounders (low income, single, and immigrant status) on theoretical grounds, based on related work in severe mental illness [35],[41], and also tested whether they were each independently associated with caseness and violent crime, respectively, in univariate analyses at the 5% level of significance [42]. In a further analysis, we additionally adjusted for comorbid substance abuse.

Power calculations (with an alpha of 0.05, and a power of 0.90) suggested that 2,385 cases and 23,850 controls were needed to determine a 1.5-fold difference in violence risk.

STROBE guidelines were followed (see Text S1 for details).

## Results

### Epilepsy

We identified 22,947 individuals with epilepsy and compared them with 224,006 age- and gender-matched general population controls (see Table 1 for baseline data). Of the cases, 973 had at least one violent conviction (4.2%) subsequent to diagnosis, compared with 5,504 controls (2.5%). We found a risk increase for violent crime in individuals with epilepsy after matching and adjustment for age, gender, and socio-demographic confounders (adjusted odds ratio [aOR] = 1.5, 95% CI: 1.4–1.7; Table 2), an absolute risk increase of 1.7% compared with age- and gender-matched population controls. This effect was attenuated after further adjustment for substance use (aOR = 1.2, 1.1–1.3). The rate for violent crime was significantly lower in those first diagnosed before age 16 y than in those first diagnosed at age 16 y or older. In addition, subtypes of epilepsy involving loss of consciousness (complex partial seizures and generalised epilepsy) were associated with lower rates of violent crime (Table 3).

**Table 1:** Baseline socio-demographic information for individuals included in the study.

Variable	Epilepsy		Traumatic Brain Injury	
	Cases (*n* = 22,947)	Unaffected General Population Controls (*n* = 224,006)	Cases (*n* = 22,914)	Unaffected General Population Controls (*n* = 229,118)
Male gender, *n* (percent)	11,965 (52.1%)	116,702 (52.1%)	16,282 (71.1%)	162,801 (71.1%)
Single status, *n* (percent)	12,965 (69.0%)	108,085 (53.3%)	12,473 (65.9%)	115,621 (55.1%)
Individual mean annual income, SEK (SD)	2,242 (1,478)	2,599 (1,374)	2,385 (1,379)	2,611 (1,416)
Mean age at diagnosis, age in years (SD)	19.8 (13.8)	n/a	24.8 (12.3)	n/a

Single status was defined as being unmarried. Data on income were from the 1990 census and were not available for 4,676 individuals with epilepsy and 53,916 matched population controls, and for 5,048 individuals with traumatic brain injury and 19,278 corresponding controls. Data on single status were not available for 4,157 individuals with epilepsy and 21,052 matched population controls, and for 3,986 individuals with traumatic brain injury and 19,278 corresponding controls. No data were missing on the other variables.

n/a, not applicable; SD, standard deviation; SEK, Swedish Kronor.

**Table 2:** Risk of violent crime in individuals after diagnosis with epilepsy or traumatic brain injury in Sweden (1973–2009) compared with general population controls.

Neurological Condition	Number of Violent Individuals (Percent)		aOR^a^ (95% CI)
	Cases	General Population Controls	
Epilepsy (*n* = 22,947)	973 (4.2%)	5,504 (2.5%)	1.5 (1.4–1.7)
Traumatic brain injury (*n* = 22,914)	2,011 (8.8%)	6,837 (3.0%)	3.3 (3.1–3.5)

a Comparison with general population controls matched by age (birth year) and gender, and adjusted by income (lowest versus middle and highest thirds), marital status (single versus not single), and immigrant status (individual born outside Sweden versus not). aOR, adjusted odds ratio.

**Table 3:** Association between epilepsy and traumatic brain injury and subsequent violent crime in Sweden (1973–2009) stratified by age of first diagnosis, clinical subtype, and severity.

Variable	Subgroup	Number of Violent Persons (Percent)	Sample Size	Statistic, *p*-Value
***Epilepsy***				
**Age of onset**	First diagnosed under age 16 y	317 (3.2%)	10,056	χ^2^_1_ = 52.1, *p*<0.001
	First diagnosed at age 16 y or older	656 (5.1%)	12,891	
**Clinical subtype**	Complex partial seizures	71 (3.1%)	2,305	χ^2^_3_ = 12.9, *p* = 0.005
	Other partial seizures	76 (5.2%)	1,474	
	Generalised	213 (3.9%)	5,453	
	Other or unspecified	519 (4.4%)	12,716	
**Severity**	Shorter treatment length (less than 15 d)	880 (4.3%)	20,548	χ^2^_1_ = 0.9, *p* = 0.35
	Longer treatment length (15 d or more)	93 (3.9%)	2,399	
***Traumatic brain injury***				
**Age of onset**	Diagnosed under age 16 y	358 (6.7%)	5,310	χ^2^_1_ = 35.7, *p*<0.001
	Diagnosed at age 16 y or older	1,653 (9.4%)	17,604	
**Clinical subtype**	Cerebral oedema	233 (7.2%)	3,234	χ^2^_2_ = 6.4, *p* = 0.04
	Focal	182 (8.9%)	2,039	
	Haemorrhagic	341 (7.1%)	4,672	
**Severity**	Traumatic brain injury	2,011 (8.8%)	22,914	χ^2^_1_ = 21.9, *p*<0.001
	Concussion only	21,078 (7.9%)	266,709	

### Traumatic Brain Injury

We compared 22,914 individuals with traumatic brain injury with 229,118 general population controls (Table 1 for baseline data), of whom 2,011 (8.8%) were violent after first diagnosis. Cases had a significantly higher risk of violent crime compared with general population controls after adjustment for age, gender, and socio-demographic confounders (aOR = 3.3, 3.1–3.5; Table 2) and further adjustment for substance abuse (aOR = 2.3, 2.2–2.5). This equated to an absolute risk increase of 5.8% in the traumatic brain injury group compared with controls.

We conducted three stratified analyses: age of injury, severity of injury, and subtypes of brain injury (Table 3). Those first diagnosed under age 16 y (versus 16 and older) and those diagnosed with concussion only (versus traumatic brain injury) had lower rates of violent crime, whereas individuals with focal injuries had higher rates than those with more diffuse or haemorrhage-related injuries.

### Sibling Control Studies

We found evidence of familial confounding in the association between both epilepsy and traumatic brain injury and subsequent violent crime. In the epilepsy group, 418 (4.0%) of the 10,360 cases had violent offenses. This was not associated with an increased odds of violent crime compared to unaffected siblings (aOR = 1.1, 0.9–1.2), where 727 out of a possible 17,448 (4.2%) individuals had violent convictions.

In the traumatic brain injury group, there were 992 (8.6%) individuals with violent offences among the 11,499 cases. This corresponded to an increased odds of violent conviction compared to unaffected siblings (aOR = 2.0, 1.8–2.3), where 832 out of a possible 19,628 (4.2%) individuals had violent convictions.

## Discussion

This population-based study examined the risk of violent crime in individuals after diagnosis with epilepsy or traumatic brain injury in Sweden over 35 y. We used longitudinal designs, adjusted for socio-demographic confounders, compared cases with both general population and unaffected sibling controls, and employed a reliable outcome (violent convictions) that allows for international comparisons. We also investigated rates of violence across diagnostic subtypes and among those with childhood-onset versus adult-onset diagnoses. With over 22,000 individuals each for the epilepsy and traumatic brain injury groups, the sample was, to our knowledge, more than 50 times larger than those used in previous related studies on epilepsy, and more than seven times larger than previous studies on brain injury [10]. Our main findings were that around 4% of individuals with epilepsy had violent convictions after first diagnosis, while approximately 9% of those with traumatic brain injury had violent convictions subsequent to diagnosis. Although this corresponded to a modest increase in the odds of violent crime in individuals with epilepsy compared to the general population, we found no risk increase in comparison with their unaffected siblings, which provided a powerful approach to adjust for familial confounding. This was in contrast to individuals with traumatic brain injury, for whom there was a 3-fold increase in the odds of violent crime compared with the general population, and there was a doubling of odds of violent crime in individuals with traumatic brain injury compared with their unaffected siblings. As these siblings shared half the genes and most of the early environment, this allowed us to partly account for personality traits that are associated with both violence and head injury or epilepsy.

For epilepsy, the findings of an absolute rate of violent crime of 4% and the lack of any association in the sibling control study should be seen in the context of expert opinion in the field that states that the link is strong [3],[8]; these findings are also potentially important with respect to the fact that epilepsy remains heavily stigmatised [20],[43],[44]. Previous views may have been influenced by high-profile criminal cases of individuals with epilepsy who committed homicide [45],[46], and reports of high prevalences of epilepsy in prisoners [11],[12], the latter that have not been subsequently confirmed [47]. Our finding on relative risks counter a recent systematic review that found a slightly protective risk for epilepsy, but this review was based on three investigations, all of which were in selected samples [10]. The finding that certain subtypes of epilepsy (including simple partial seizures and temporal lobe epilepsy) are associated with higher rates of violent crime may assist in clarifying mechanisms and potential treatments, and suggests that these patients could be assessed for violence risk if these findings on subtypes are validated. Frontal lobe seizures, associated with violence in some cases [48], could be one mechanism to explain the excess in simple partial seizures. Interestingly, these subgroup differences are consistent with the finding for traumatic brain injury, as discussed below, that focal, in contrast to generalised, brain injuries were linked with higher violence risk.

The increased risk of violent crime in individuals with traumatic brain injury compared with general population controls is consistent with clinical studies [39],[49]–[52] and a recent systematic review [10]. However, the latter review identified no population-based or unselected investigations, and synthesised information based on around 2,500 individuals with head injury. This previous review reported a risk increase for brain injury as a pooled odds ratio of 1.7. The current report findings nearly double this risk estimate, even after adjustment for socio-demographic confounders. As there is likely to be residual confounding in such comparisons, we used unaffected siblings as controls, and found a moderated but still significant association with violent crime. Therefore, although there are plausible aetiological hypotheses that propose mechanisms for violence in individuals with traumatic brain injury, including damage to the frontal and prefrontal cortices [14],[53], this study suggests that shared familial factors explain some of this association. Familial confounding may occur through genetic susceptibility, early environmental effects, or both. Such effects may involve personality traits (such as impulsivity, risk taking, and propensity to substance abuse [54]) and handling of interpersonal situations that increase the risk of head injury and are also associated with violence.

Despite evidence of familial confounding, we found support for a direct effect in brain injury leading to violence, in that focal brain injuries were associated with the highest risk, although the diagnostic information available in the hospital registers did not specify the location of the injury. Regarding the finding that younger age was related with less likelihood of subsequent violent crime, one explanation might be that earlier injuries are associated with better outcomes because of neuroplasticity or more effective treatments [55], or that a later onset of injury is more strongly correlated with an antisocial lifestyle, sensation seeking, and risk taking. Further research is necessary to identify the specific mechanisms underlying the age effect and familial confounding, and may contribute to the development of preventive strategies. Examining the role of repetitive brain injuries on risk of violence is another area where further research is necessary. Other work has found some evidence that cumulative mild injuries might lead to a longer period of future antisociality [56] and increased risk of repeat offending in prisoners [17],[57].

From a public health and policy perspective, these findings point in different directions for health services, the criminal justice system, and patient charities. On the one hand, the lack of an association with violent crime in epilepsy cases after adjustment for familial factors may be valuable for patient charities and other stakeholders in addressing one of the potential causes of the stigma associated with this condition. Health services may consider violence risk assessment and management worthwhile in certain high-risk groups of individuals with epilepsy, particularly if they have violent histories. For traumatic brain injury, absolute and relative risks more clearly suggest that there are certain groups of patients who would benefit from violence risk assessment. As current guidelines for the assessment of brain injury make no recommendations in relation to the assessment or investigation of violence risk [58], our findings suggest that these may need review, at least for some groups of patients with traumatic head injury, particularly if they abuse illegal drugs or alcohol. In prisoners with traumatic head injury, improved screening, assessment, and management may improve repeat offending rates [17]. An additional group that may benefit from more detailed assessment and treatment are head injured juvenile delinquents [59]. The odds ratio of violent crime reported in this study for head injury (3.3) is similar to those reported for schizophrenia (where violence risk assessment should be routinely considered [60]) and bipolar disorder [41], but less than the odds ratio of 7–9 reported for substance abuse [1].

### Limitations

Study weaknesses include our reliance on patient registers for case ascertainment. This meant that the sample was selected towards more severe cases of epilepsy and traumatic brain injury. This could have led to an underestimation of the association with violent crime if individuals with more severe disease are more likely to be physically disabled and thus less likely to commit violent crime (although the use of outpatient information should have moderated against this). However, it is also possible that the more severe presentations of these disorders are more prone to violence, and hence we may have overestimated the risk. We found some support for this in persons with traumatic brain injury, who had higher rates of violent crime than those with concussion diagnoses alone. The fact that we may have oversampled the more severe cases may be more relevant in epilepsy, as we selected only individuals with two or more hospital diagnoses of epilepsy in order to improve diagnostic specificity, but the reported finding that those with longer treatment episodes were not at higher risk of violent crime argues against this potential bias. Nevertheless, as we are not certain what proportion of individuals with epilepsy are hospitalised over a 30-y period, our results may be less generalisable to individuals with epilepsy who are not inpatients or outpatients at some point in their illness. Another limitation was the lack of data on the extent and character of treatment for these conditions. It is possible that treatment effects mediated some of the differences found here, particularly the mood-stabilising effects of anticonvulsants prescribed to epilepsy patients, although a recent review found no clear evidence that such medications reduce violence [61]. In the analyses, we adjusted for substance abuse but did not examine comorbidity for other psychiatric illnesses, as was done in one Danish population study [62], since the validity of outpatient data for less severe mental illnesses, such as depression and anxiety disorders, is uncertain. Nevertheless, future work could examine whether the risk differs by comorbidity. Although we relied on conviction data, other work has shown that the degree of underestimation of violence is similar in psychiatric patients and controls compared with self-report measures, and hence the risk estimates were unlikely to be affected [63]. This has also been found for studies investigating violence risk in individuals with schizophrenia [1]. We have no reason to think that this would be different for these two neurological conditions. Overall rates of violent crime and their resolution are mostly similar across western Europe, suggesting some generalisability of our findings [64]. Comparisons with the US are more difficult because of differences in legal and judicial systems, but police-recorded assault rates for the time period 1981–1999 were 3.7 per 1,000 individuals in the US and 4.1 per 1,000 individuals in Sweden [65].

In conclusion, by using Swedish population-based registers over 35 y, we reported risks for violent crime in individuals with epilepsy and traumatic brain injury that contrasted with each other, and appeared to differ within each diagnosis by subtype, severity, and age at diagnosis. The implications of these findings are likely to vary for clinical services, the criminal justice system, and patient charities.

## Supporting Information

Text S1 **STROBE statement checklist of items that should be included in reports of observational studies.**
(DOCX)

## Abbreviations

aOR: adjusted odds ratio
ICD: International Classification of Diseases
