# Supplementary material for: Risk of Violent Crime in Individuals with Epilepsy and Traumatic Brain Injury: A 35-Year Swedish Population Study
Source: PLoS Med. 2011 Dec 27;8(12):e1001150. doi: 10.1371/journal.pmed.1001150 (PMC3246446; doi:10.1371/journal.pmed.1001150)
Supplement: Text S1 — STROBE statement checklist of items that should be included in reports of observational studies. (DOCX) [file pmed.1001150.s001.docx]

**Text S1 - STROBE statement checklist of items that should be included in reports of observational studies**

**Title and abstract**

1. (a) Indicate the study's design with a commonly used term in the title or the abstract

DONE – abstract indicates that this a study of cases and controls from Swedish population registers followed up longitudinally for violent crime. The best description of the study design is a matched cohort.

(b) Provide in the abstract an informative and balanced summary of what was done and what was found

DONE

**Introduction**

Background/rationale

2 Explain the scientific background and rationale for the investigation being reported

DONE (paragraphs 1-2)

Objectives

3 State specific objectives, including any prespecified hypotheses

DONE (paragraph 2)

**Methods**

Study design

4 Present key elements of study design early in the paper

DONE (paragraph 4)

Setting

5 Describe the setting, locations, and relevant dates, including periods of recruitment, exposure, follow-up, and data collection

DONE (paragraphs 4-5)

Participants

6 (a) Case-control study - Give the eligibility criteria, and the sources and methods of case ascertainment and control selection. Give the rationale for the choice of cases and controls

DONE (paragraphs 5, 7, 8) – although a more accurate description of the design is that of a matched cohort.

(b) Case-control study - For matched studies, give matching criteria and the number of controls per case

DONE (paragraph 7)

Variables

7 Clearly define all outcomes, exposures, predictors, potential confounders, and effect modifiers. Give diagnostic criteria, if applicable

DONE (paragraph 9 for outcomes, paragraphs 5 and 6 for diagnostic criteria)

Data sources/ measurement

8 For each variable of interest, give sources of data and details of methods of assessment (measurement). Describe comparability of assessment methods if there is more than one group

DONE (paragraphs 8 and 10)

Bias

9 Describe any efforts to address potential sources of bias

DONE (paragraph 12)

Study size

10 Explain how the study size was arrived at

DONE (paragraph 13)

Quantitative variables

11 Explain how quantitative variables were handled in the analyses. If applicable, describe which groupings were chosen and why

DONE (paragraph 7)

Statistical methods

12 (a) Describe all statistical methods, including those used to control for confounding

DONE (paragraphs 8 and 12)

(b) Describe any methods used to examine subgroups and interactions

DONE (paragraph 8)

(c) Explain how missing data were addressed

DONE (paragraph 11)

(d) Case-control study -If applicable, explain how matching of cases and controls was addressed

DONE (paragraph 12)

(e) Describe any sensitivity analyses

DONE (paragraphs 7 and 12)

**Results**

Participants

1. (a) Report numbers of individuals at each stage of study- eg numbers potentially eligible, examined for eligibility, confirmed eligible, included in the study, completing follow-up, and analysed

DONE

1. Give reasons for non-participation at each stage N/A (total population study)

(c) Consider use of a flow diagram N/A

Descriptive data

14 (a) Give characteristics of study participants (eg demographic, clinical, social) and information on exposures and potential confounders

DONE (table 1, and Results section)

1. Indicate number of participants with missing data for each variable of interest

DONE (see notes to table 1)

(c) Cohort study - Summarise follow-up time (eg average and total amount) N/A

Outcome data

15 Case-control study - Report numbers in each exposure category, or summary measures of exposure

DONE

Main results

16 (a) Report the numbers of individuals at each stage of the study - eg numbers potentially eligible, examined for eligibility, confirmed eligible, included in the study, completing follow-up, and analysed

DONE

(b) Give reasons for non-participation at each stage N/A

(c) Consider use of a flow diagram N/A

Other analyses

17 Report other analyses done - eg analyses of subgroups and interactions, and sensitivity analyses

DONE (see table 3)

**Discussion**

Key results

18 Summarise key results with reference to study objectives

DONE (paragraph 19)

Limitations

19 Discuss limitations of the study, taking into account sources of potential bias or imprecision. Discuss both direction and magnitude of any potential bias

DONE (paragraph 24)

Interpretation

20 Give a cautious overall interpretation of results considering objectives, limitations, multiplicity of analyses, results from similar studies, and other relevant evidence

DONE

Generalisability

21 Discuss the generalisability (external validity) of the study results

DONE under Limitation section (paragraph 24)

**Other information**

Funding

22 Give the source of funding and the role of the funders for the present study and, if applicable, for the original study on which the present article is based

DONE – The funders had no role in any aspect of the study design, conduct or interpretation.
